# Exosomal mRNA Signatures as Predictive Biomarkers for Risk and Age of Onset in Alzheimer’s Disease

**DOI:** 10.3390/ijms252212293

**Published:** 2024-11-15

**Authors:** Daniel A. Bolívar, María I. Mosquera-Heredia, Oscar M. Vidal, Ernesto Barceló, Ricardo Allegri, Luis C. Morales, Carlos Silvera-Redondo, Mauricio Arcos-Burgos, Pilar Garavito-Galofre, Jorge I. Vélez

**Affiliations:** 1 Department of Industrial Engineering, Universidad del Norte, Barranquilla 081007, Colombia; 2 Department of Medicine, Universidad del Norte, Barranquilla 081007, Colombia; 3 Instituto Colombiano de Neuropedagogía, Barranquilla 080020, Colombia; 4 Department of Health Sciences, Universidad de La Costa, Barranquilla 080002, Colombia; 5 Grupo Internacional de Investigación Neuro-Conductual (GIINCO), Universidad de La Costa, Barranquilla 080002, Colombia; 6 Institute for Neurological Research FLENI, Montañeses 2325, Buenos Aires C1428AQK, Argentina; 7 Grupo de Investigación en Psiquiatría (GIPSI), Departamento de Psiquiatría, Instituto de Investigaciones Médicas, Facultad de Medicina, Universidad de Antioquia, Medellín 050010, Colombia; mauricio.arcos@udea.edu.co

**Keywords:** Alzheimer’s disease, exosomes, mRNA, machine learning, personalized medicine

## Abstract

Alzheimer’s disease (AD) is a neurodegenerative disorder characterized by progressive cognitive decline and memory loss. While the precise causes of AD remain unclear, emerging evidence suggests that messenger RNA (mRNA) dysregulation contributes to AD pathology and risk. This study examined exosomal mRNA expression profiles of 15 individuals diagnosed with AD and 15 healthy controls from Barranquilla, Colombia. Utilizing advanced bioinformatics and machine learning (ML) techniques, we identified differentially expressed mRNAs and assessed their predictive power for AD diagnosis and AD age of onset (ADAOO). Our results showed that ENST00000331581 (*CADM1*) and ENST00000382258 (*TNFRSF19*) were significantly upregulated in AD patients. Key predictors for AD diagnosis included ENST00000311550 (*GABRB3*), ENST00000278765 (*GGTLC1*), ENST00000331581 (*CADM1*), ENST00000372572 (*FOXJ3*), and ENST00000636358 (*ACY1*), achieving > 90% accuracy in both training and testing datasets. For ADAOO, ENST00000340552 (*LIMK2*) expression correlated with a delay of ~12.6 years, while ENST00000304677 (*RNASE6*), ENST00000640218 (*HNRNPU*), ENST00000602017 (*PPP5D1*), ENST00000224950 (*STN1*), and ENST00000322088 (*PPP2R1A*) emerged as the most important predictors. ENST00000304677 (*RNASE6*) and ENST00000602017 (*PPP5D1*) showed promising predictive accuracy in unseen data. These findings suggest that mRNA expression profiles may serve as effective biomarkers for AD diagnosis and ADAOO, providing a cost-efficient and minimally invasive tool for early detection and monitoring. Further research is needed to validate these results in larger, diverse cohorts and explore the biological roles of the identified mRNAs in AD pathogenesis.

## 1. Introduction

Alzheimer’s disease (AD), the most common form of dementia [1], originates from a combination of genetic, environmental, and lifestyle factors that contribute to the accumulation of amyloid-beta (Aβ) plaques and hyperphosphorylated tau tangles in the brain [2,3,4,5]. Although aging is the primary risk factor of late-onset AD (>65 y/o) [3], alleles harbored in major and minor effect genes play a significant role in shaping the architecture of AD etiology [5,6]. Currently, AD diagnosis involves a combination of cognitive assessments, brain imaging, and biomarker analysis. However, early detection of AD remains elusive due to the subtle nature of early symptoms [5,7,8,9,10,11,12,13,14].

Messenger RNA (mRNA) transcripts are single-stranded RNA molecules that serve as intermediates between the genetic information encoded in DNA and the synthesis of proteins via translation. Analysis of brain mRNA expression has allowed researchers to identify differences between individuals with AD and healthy controls, as well as genes actively involved in AD development and progression [15,16]. This provides valuable insights into the molecular pathways and cellular processes that are dysregulated in the disease [15,17].

One significant breakthrough in AD detection has been the identification of the SRSF1 and PTBP1 proteins in regulating AD-related genes [18]. These proteins act as splicing factors, influencing the production of specific isoforms of the *CD33* gene, which is associated with AD. Other studies linked mRNA expression for specific genes, such as the acetylcholinesterase (*ACHE*) gene, proposed as a potential biomarker for diagnosing AD and related conditions [19]. This association is crucial as it links mRNA expression to oxidative stress, a key contributor to the progression of AD.

Additionally, cellular hypoxia can influence AD development by altering pre-mRNA splicing, particularly of the Tau gene, suggesting that environmental influences can significantly impact AD progression through changes in mRNA processing [20]. Furthermore, crucial microRNA-mRNA pairs, such as miR-26a-5p/PTGS2, have been identified as essential regulators in AD, highlighting the importance of regulatory networks and post-transcriptional regulation in AD development [21,22]. Recently, the microRNA 221, which is a cerebrospinal fluid microRNA, has emerged as a promising candidate for the early detection of AD [6,23,24], suggesting that the study of mRNA has the potential to advance the development of new diagnostic tools and therapeutic strategies for AD [25,26].

Since 2020, a collaborative effort has been underway to elucidate the genetic landscape of AD susceptibility and AD age of onset (ADAOO) in Barranquilla, Colombia. This involves a comprehensive clinical, cognitive, neuropsychological, and genetic assessment of individuals with AD (cases) and healthy unrelated controls. In this report, we present the results and analysis of microarrays quantifying the expression of 16,580 mRNAs using advanced bioinformatics, data analytics, and ML techniques to identify exosomal mRNA signatures that could improve disease diagnosis, prediction, and treatment. We hypothesize that (1) mRNAs could be promising, non-invasive, and reliable novel diagnostic markers for AD in this population, and (2) these mRNA signatures could potentially allow early diagnosis, risk prediction, and the development of targeted interventions for this devastating neurodegenerative disease. We identify differentially expressed mRNAs that could serve as potential biomarkers for AD diagnosis and ADAOO. Our results suggest that integrating mRNAs with ML tools could improve early detection and monitoring. Additionally, we provide insights into the role of mRNAs within the *CADM1* and *TNFRSF19* genes in AD pathology. While our findings are promising, further validation in larger cohorts is essential to confirm the reliability of these biomarkers and explore their roles in disease mechanisms.

## 2. Results

### 2.1. Subjects

We collected data from 30 individuals through clinical evaluations, family histories, comprehensive neurological and neuropsychological clinical examinations, and structured interviews. Demographic data are summarized in Table 1. The Universidad del Norte Ethics Committee approved this study (Project Approval Act #188 of 23 May 2019).

### 2.2. mRNA Signatures Contributing to AD Susceptibility via Logistic Regression

**Figure 1:**
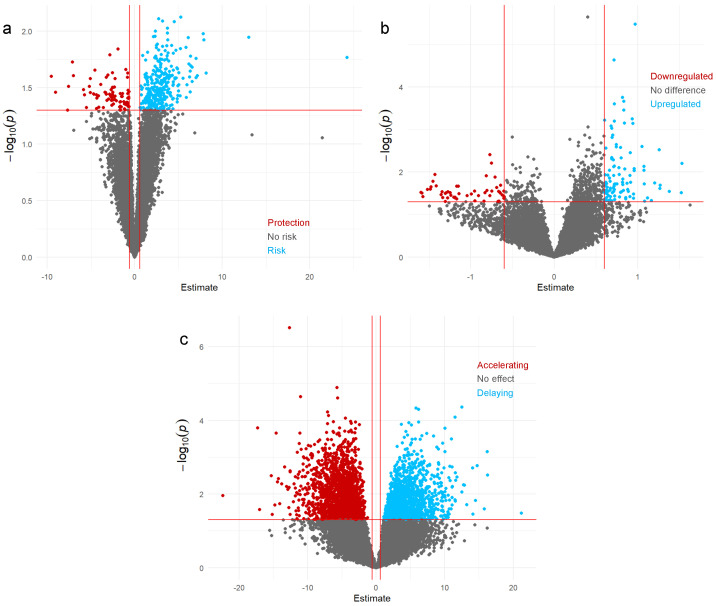
Volcano plots for mRNAs (**a**) conferring AD susceptibility, (**b**) differentially expressed mRNAs between the comparison groups, and (**c**) associated with ADAOO. Red lines show statistically significant mRNAs at 5%.

The expression of 16,580 mRNAs was quantified in all participants, identifying 385 significantly associated with AD at a 5% nominal level. Of these, 82 mRNAs had a protective effect against AD, while 303 mRNAs were associated with an increased risk of an AD diagnosis (Figure 1a). However, none of these mRNAs were statistically significant after FDR correction.

**Figure 2:**
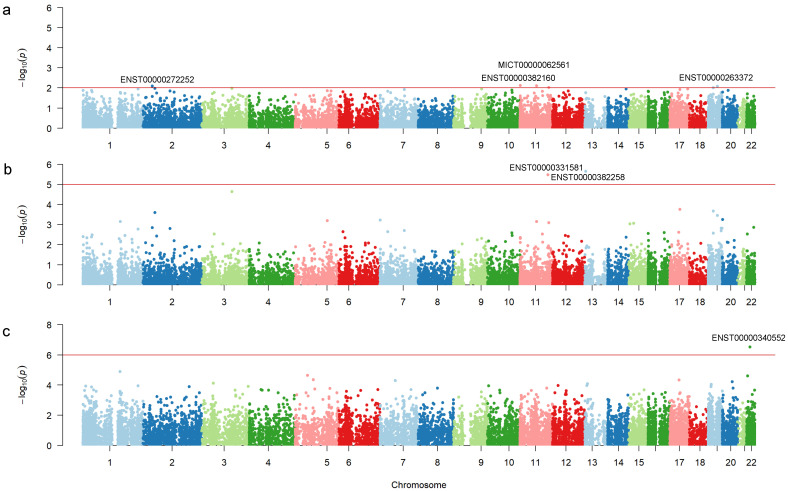
Manhattan plots showing mRNA signatures (**a**) conferring susceptibility to AD (*p* < 0.01 threshold, red line), (**b**) differentially expressed between study groups (*p* < 2.5 × 10^−6^ threshold, red line), and (**c**) associated with ADAOO (*p* < 2.5 × 10^−6^ threshold, red line) in a sample of 15 individuals with AD from Barranquilla, Colombia.

Table 2 shows the top 10 mRNAs conferring susceptibility to AD in our sample, which are in the *KRTAP5-6*, *TPCN2*, *GALM*, *KCNK6*, *CXCR5*, *ZNF626*, *STON1*, *C3orf22*, *AKNA*, and *SMIM5* genes. Figure 2a shows the *p*-value distribution across chromosomes. Note that mRNAs most significantly associated with AD susceptibility are in chromosomes 2, 11, and 19.

### 2.3. mRNAs Signatures Differentially Expressed Between Comparison Groups

We identified 154 differentially expressed mRNAs using Gamma regression with a Type I error of 5%; 102 mRNAs were upregulated, and 52 were downregulated in individuals with AD compared to healthy controls (Figure 1b). Table 3 shows the top 10 differentially expressed mRNAs in our sample, and Figure 2b shows the distribution of *p*-values across chromosomes. However, only ENST00000331581 (*CADM1*) and ENST00000382258 (*TNFRSF19*) were statistically significantly differentially expressed after FDR correction.

### 2.4. mRNAs Signatures Modifying ADAOO

We identified 2034 mRNAs that had a delaying effect on ADAOO ($\hat{\beta }$ > 0) and 1468 mRNAs that accelerated ADAOO ($\hat{\beta }$ < 0) in our individuals with AD, with a nominal Type I error of 5%. Table 4 shows the top 10 mRNAs associated with ADAOO in our sample. Interestingly, ENST00000257696 ($\hat{\beta }=4.34$; *HILPDA*) and ENST00000304060 ($\hat{\beta }=4.79$; *ZNF440*) delay ADAOO, whereas ENST00000263851 ($\hat{\beta }=-17.31$; *IL7*), ENST00000340552 ($\hat{\beta }=-12.6$; *LIMK2*), and ENST00000230658 ($\hat{\beta }=11.05$; *ISL1*) are the top accelerators. However, only ENST00000340552 (*LIMK2*), which accelerates AD onset by ~12.6 years (Table 4) showed a statistically significant association with ADAOO after FDR correction.

### 2.5. mRNAs Signatures Identified via ML

We identified several mRNAs with high accuracy for predicting AD diagnosis and ADAOO using the one-rule (OneR) ML algorithm (Table 5). Notably, the ENST00000331581 (*CADM1*), ENST00000372572 (*FOXJ3*) and ENST00000311550 (*GABRB3*) mRNAs independently achieved an accuracy of 95.4% for predicting AD diagnosis in the training dataset (*n =* 21). Regarding ADAOO, ENST00000640218 (*HNRNPU*), ENST00000261245 (*MNAT1*), and ENST00000339562 (*NR4A2*) exhibited a remarkable ability to accurately predict ADAOO in the training dataset (*n* = 11; Table 5).

### 2.6. ML-Based Predictive Framework of AD Diagnosis

**Figure 3:**
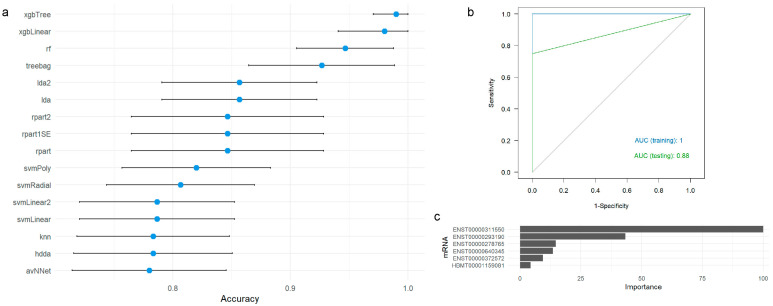
(**a**) Accuracy and 95% confidence intervals for predicting AD diagnosis using different ML algorithms based on the top 30 mRNAs identified with OneR. (**b**) ROC curves for the xgbTree algorithm in the training (blue) and testing (green) datasets. (**c**) Variable importance analysis for the xgbTree algorithm. ROC: receiver operating characteristic; AUC: area under the ROC curve.

We evaluated the performance of several ML algorithms to construct a predictive framework for AD diagnosis based on the 30 mRNAs with the highest predictive power identified via the OneR ML algorithm (Supplementary Table S2). Figure 3a summarizes their accuracy in the training dataset.

Our findings show that the rf, xgbLinear, and xgbTree ML perform exceptionally well in predicting AD diagnosis based on mRNA expression levels, achieving accuracies of 94.7%, 98%, and 99%, respectively (Table 6). Notably, the xgbTree algorithm exhibits low standard deviation and coefficient of variation. In contrast, the avNNet, hdda, and knn algorithms showed lower accuracy and more significant variability.

Further evaluation of the xgbTree algorithm confirmed its robust predictive capability for AD diagnosis (Table 7). Analysis of the ROC curve and AUC for the xgbTree algorithm across training and testing datasets suggest that this ML algorithm is capable of distinguishing individuals with AD from healthy controls and that ENST00000311550 (*GABRB3*) is the most significant mRNA for predicting AD (Supplementary Figure S1).

### 2.7. Feature Selection and Model Refinement for AD Diagnosis

We applied the OneR algorithm to enhance our ML-based approach for AD diagnosis and narrowed the predictors to the top five mRNAs. Our analysis identified that ENST00000311550 (*GABRB3*), ENST00000278765 (*GGTLC1*), ENST00000331581 (*CADM1*), ENST00000372572 (*FOXJ3*), and ENST00000636358 (*ACY1*) have the highest predictive power for AD diagnosis.

Subsequently, we assessed the performance of different ML algorithms based on these mRNAs (Supplementary Table S4). Interestingly, some ML algorithms achieved remarkable accuracy scores (i.e., avNNet, lda, lda2, svmLinear, svmLinear2, svmPoly, treebag, xgbLinear, and xgbTree), while others, despite showing slightly lower accuracies and higher variability, perform reasonably well (i.e., svmRadial, knn, and rf). This suggests that ML algorithms using the top 5 mRNAs identified via OneR can distinguish between individuals with AD and healthy controls in our sample. However, the xgbTree algorithm is the preferred choice. This model achieves remarkable performance in training and testing datasets, with the ROC curve and AUC values indicating that the ML-based model is robust, generalizable, and capable of accurately distinguishing AD individuals from healthy controls (Supplementary Figure S1). The ENST00000311550 (*GABRB3*) mRNA is the most important predictor.

To further enhance the prediction accuracy for AD diagnosis, we explored combinations of mRNAs when using the xgbTree ML algorithm. Our goal was to identify the most effective predictors for diagnosing AD. Thus, we assessed the predictive power of eight pairs of gene transcripts. Of these, the pair ENST00000311550 (*GABRB3*) and ENST00000331581 (*CADM1*) emerged as the most accurate, achieving an average accuracy of 95.8% in the training data (Supplementary Figure S2). Variable importance revealed that, under this model, ENST00000311550 (*GABRB3*) is the most important predictor for AD diagnosis. ENST00000278765 (*GGTLC1*) was included as a predictor for the final predictive model because it also demonstrated good predictive power and robust performance metrics. The final model with these three mRNAs achieved remarkable AUC, accuracy, sensitivity, specificity, and precision scores during the training and testing phases (Supplementary Figure S2).

### 2.8. ML-Based Predictive Framework for ADAOO

Table 8 reports the performance of several ML algorithms for predicting ADAOO based on the top 30 mRNAs identified via OneR (Table S3, Supplementary Material). Our results indicate that the pls and known algorithms demonstrated superior performance; the former achieved an RMSE of 6.519 and an MAE of 6.459, while the known achieved RMSE and MAE values of 6.817 and 6.761, respectively.

**Figure 4:**
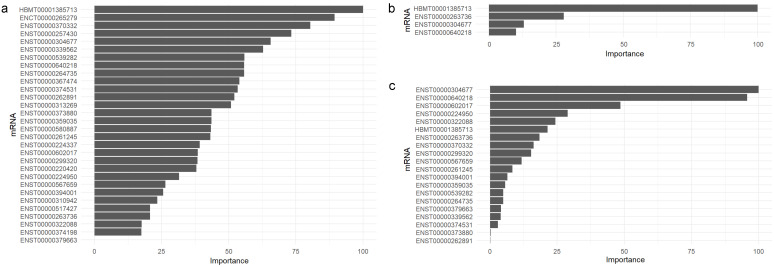
Variable importance for the (**a**) rf, (**b**) xgbLinear, and (**c**) xgbTree ML algorithms for predicting ADAOO. Here, higher values are better.

ML algorithms were clustered into three groups (Supplementary Figure S3). Variable importance analyses of the top performer algorithms revealed distinct prioritizations for predicting ADAOO. For instance, HBMT00001385713 (*LONRF1*), ENCT00000265279 (*INSM1*), ENST00000370332 (*GFI1*), and ENST00000257430 (*APC*) are pivotal variables for ADAOO prediction using rf (Figure 4a); HBMT00001385713 (*LONRF1*), ENST00000263736 (*SRBD1*), ENST00000304677 (*RNASE6*), and ENST00000640218 (*HNRNPU*) are identified as the most important by the xgbLinear algorithm (Figure 4b); and xgbTree ranks ENST00000304677 (*RNASE6*), ENST00000640218 (*HNRNPU*), ENST00000602017 (*PPP5D1*), ENST00000224950 (*STN1*), and ENST00000322088 (*PPP2R1A*) mRNAs as the most critical ADAOO predictors in our cohort of individuals with AD (Figure 4c).

### 2.9. Refining the ML-Based Model for ADAOO Prediction

We selected the top five mRNAs to construct pair combinations and tested their ADAOO predictive power for the testing dataset using the rf, xgbLinear, and xgbTree algorithms (Table 9). Among the distinct model combinations, the xgbTree algorithm with ENST00000304677 (*RNASE6*) and ENST00000602017 (*PPP5D1*) as predictors achieved the best performance (RMSE = 0.462, *R*^2^ = 0.993, MAE = 0.392; Table 9).

## 3. Discussion

This study explored the utility of various data analytics and machine learning (ML) techniques for identifying patterns in mRNA expression data related to Alzheimer’s disease (AD). The key findings are as follows: ML methods successfully identified differentially expressed mRNAs in AD, providing insights into their roles in disease pathogenesis. Logistic regression analysis revealed 385 differentially expressed mRNAs, 82 showing a protective effect and 303 associated with increased AD risk. Secondly, an ML-based framework predicts AD based on mRNA profiles, demonstrating promise for early detection and personalized intervention strategies. Several mRNA transcripts, including ENST00000331581 (*CADM1*), ENST00000372572 (*FOXJ3*), and ENST00000311550 (*GABRB3*), exhibited exceptional predictive power, accurately distinguishing AD cases from controls. Lastly, the study extended to predicting the age of AD onset (ADAOO), highlighting the potential for personalized treatment planning based on individual risk assessments. Key mRNA transcripts, such as ENST00000304677 (*RNASE6*) and ENST00000602017 (*INPP5D*), were identified as crucial predictors of ADAOO by advanced ML algorithms such as xgbTree and RF [27,28].

Logistic regression analysis revealed 385 differentially expressed mRNAs, with 82 demonstrating a protective effect and 303 associated with an increased risk of AD. Although these mRNAs did not reach statistical significance (Table 2), the biological relevance of these mRNAs could play an important role in AD pathogenesis.

We used Gamma regression to identify differentially expressed mRNAs and their association with AD and ADAOO as complementary analyses. A total of 154 differentially expressed mRNAs, 102 upregulated and 52 downregulated in individuals with AD, were identified. Of these, two mRNAs, ENST00000331581 (*CADM1*) and ENST00000382258 (*TNFRSF19*), were statistically significant after multiple testing corrections were applied (Table 3). These mRNAs provide supporting evidence of the role of the *CADM1* and *TNFRSF19* in AD pathogenesis. Interestingly, *CADM1* is implicated in synaptic assembly and has known isoforms, such as SP3, identified in proteogenomic studies [29,30]. The detection of these isoforms in humans and mice, along with their altered expression in AD models, highlights their potential role in neurodegenerative processes [29].

In our study, an mRNA within the *TNFRSF19* (*TROY*) gene also emerged as an important biomarker. Previous studies have linked TNFRSF19 elevated expression to both intracranial aneurysms and coronary artery disease [31,32]. Furthermore, its strong correlation with inflammatory markers and immune-related genes highlights its potential role in chronic inflammation and vascular abnormalities. Indeed, elevated expression of *CADM1* and *TNFRSF19* in AD models emphasizes their critical role in inflammatory processes associated with AD [31,32].

We identified that expression levels in ENST00000340552 (*LIMK2*) delay ADAOO by ~12 years (Table 4). LIMK2 is a protein crucial for controlling the dynamics of the cell’s internal framework, known as the actin cytoskeleton. This process is vital for shaping cell structure and movement. When activated by ROCK1, LIMK2 can modify another protein called cofilin, which normally destabilizes the actin network. By phosphorylating cofilin, LIMK2 prevents it from breaking down actin, allowing cells to maintain their shape and move effectively. This regulation of actin is essential for fundamental cellular activities like cell division, apoptosis, and cell migration [33]. Researchers have also linked abnormalities in the *ROCK1*/*LIMK2*/cofilin pathway to various types of cancer [34,35]. *LIMK2* is also involved in neurodevelopmental disorders and neurodegenerative diseases, including AD, Parkinson’s, and schizophrenia [36]. Recent studies show that targeting *LIMK2* in cancer and neurological disorders is promising, as LIMK2 inhibitors have shown efficacy in preclinical models [37,38,39]. Thus, identifying an mRNA regulating *LIMK2* as significantly associated with delayed ADAOO emphasizes its potential as a neuroprotective factor. Given its role in actin dynamics and broad impact on cellular processes, *LIMK2* represents a valuable target for therapeutic strategies aimed at delaying the onset or progression of AD.

Using the OneR ML algorithm, we identified that transcripts ENST00000331581 (*CADM1*), ENST00000372572 (*FOXJ3*), and ENST00000311550 (*GABRB3*) each achieved an accuracy of 95.4% for distinguishing AD cases from controls (Table 5) [36,40,41,42,43]. Similarly, the performance of an ML-based predictive framework for AD diagnosis using 16 ML algorithms and the expression levels of several mRNAs was rigorously evaluated and assessed (Figure 3). Notably, RF, xgbLinear, and xgbTree emerged as top performers (Table 6). The subsequent application of the OneR ML algorithm further refined our predictive approach by identifying five key mRNA transcripts—ENST00000311550, ENST00000278765, ENST00000331581, ENST00000372572, and ENST00000636358 (Supplementary Figures S1 and S2). The fact that the gene regulated by ENST00000311550 is particularly involved in critical biological processes related to neurodegeneration and synaptic function underscores its potential as a key biomarker for AD diagnosis [44,45,46]. Analysis of GABAergic signaling components in post-mortem human brain tissue revealed significant transcriptional downregulation of *GABA* receptors (*GABBR2*, *GABRA1*, *GABRB3*, *GABRG2*), *GABA* synthesizing enzymes (GAD1, GAD2), and other neurotransmitter receptors (GRIK1, GRIK2), implicating a disruption in the excitatory/inhibitory balance that contributes to cognitive decline in AD [44]. These findings align with previous studies linking alterations in GABAergic pathways to AD pathology, suggesting potential therapeutic targets aimed at restoring neuronal function through modulation of these pathways [46]. Moreover, insights from genetic studies in epilepsy highlight parallels in synaptic dysfunction, reinforcing the broader implications of disrupted neuronal networks in neurodegenerative diseases like AD [45,46]. In addition, genes regulated by ENST00000331581, ENST00000372572, and ENST00000636358 are implicated in essential processes such as cell adhesion, synaptic function, and neuronal signaling, which are crucial in the context of neurodegenerative diseases like AD [36,41,43,47,48].

We developed and comprehensively assessed the performance of an ML-based framework for predicting ADAOO (Table 8). Among the algorithms tested, avNNet exhibited poor performance metrics, whereas knn, pls, xgbTree, xgbLinear, and RF consistently demonstrated superior predictive accuracy with low RMSE and MAE values (Table 8). Notably, the xgbTree algorithm exhibited exceptional performance in predicting ADAOO, with an impressively low RMSE and high *R*^2^ values (Table 9). Key mRNA transcripts such as ENST00000304677 and ENST00000602017 were identified as pivotal for predicting ADAOO by xgbTree, which suggests their critical role in delineating ADAOO in our sample (Table 9). ENST00000304677, located within the *RNASE6* gene, plays an important role in innate immune responses and has been linked to neuroinflammation, a characteristic feature of AD. Specifically, *RNASE6* expression correlates with myeloid-derived suppressor cells (MDSCs), suggesting its involvement in immune modulation that may influence susceptibility to ADAOO. *RNASE6* expression interacts with *APOE-ε4* status, indicating that higher levels of *RNASE6* are associated with poorer memory outcomes among *APOE-ε4* carriers [27,49]. *RNASE6* encodes an antimicrobial peptide involved in innate immune responses and has been identified in gene co-expression networks with other inflammatory genes implicated in AD, such as *TREM2* and *MS4A* [50,51].

Furthermore, ENST00000602017, identified as crucial in the predictive model, regulates the Inositol polyphosphate-5-phosphatase (*INPP5D*) gene, also known as *SHIP1*, which has emerged as significant in AD pathophysiology, particularly associated with late-onset AD (LOAD). *INPP5D* is selectively expressed in brain microglia and has been linked to LOAD through genome-wide association studies [28]. Despite its critical role, the precise impact of *INPP5D* on disease onset and progression remains unclear. Differential gene expression analysis investigated *INPP5D* in AD, revealing its upregulation in LOAD and positive correlation with amyloid plaque density. In the 5xFAD amyloid mouse model, *INPP5D* expression increased with disease progression, particularly in plaque-associated microglia. Notably, depletion of microglia using the colony-stimulating factor receptor-1 antagonist PLX5622 entirely abolished the elevated Inpp5d expression levels in 5xFAD mice.

Similarly, RF revealed the significance of HBMT00001385713 and ENST00000257430 in the ML-based predictive models of ADAOO (Table 9). ENST00000257430, associated with the APC/C-Cdh1 pathway, plays a crucial role in AD pathophysiology [52]. The APC/C-Cdh1 complex, an E3 ubiquitin ligase, regulates synaptic plasticity and neuronal survival. In AD, aberrant activation of Aβ induces phosphorylation of Cdh1, disrupting the APC/C-Cdh1 complex. This disruption leads to the accumulation of substrates such as Rock2 and Cyclin B1 in affected brain regions, contributing to synaptic loss and neurotoxicity. Studies in neurons and animal models have demonstrated that maintaining normal APC/C-Cdh1 activity may mitigate Aβ-induced neurotoxic effects, suggesting potential therapeutic targets for AD [53].

In summary, our study showed that using ML algorithms to assess AD risk and ADAOO based on demographic and genetic data is promising for clinical applications, as indicated by the RMSE, MAE, and *R*^2^ performance metrics. Genetic variants are essential predictors in our ML models for AD and ADAOO. These models can facilitate personalized assessments, ultimately advancing predictive genomics and personalized medicine approaches for AD and improving individualized treatment strategies for patients at risk of developing the disease [54,55,56]. Thus, integrating mRNA biomarkers with advanced ML methods shows potential for early ADAOO detection and intervention, enhancing clinical management strategies and improving our understanding and treatment of AD.

Integrating ML and mRNA data in AD research presents a robust framework for advancing our understanding of the disease [54,55,56]. Identified mRNAs associated with AD risk, protection, and ADAOO prediction establish a solid foundation for future investigations, particularly in Latin American and Caribbean regions [6,26,57]. Validating these findings in more extensive, diverse cohorts and exploring the biological roles of the identified mRNAs could unveil novel insights into AD pathogenesis. Furthermore, these findings hold significant therapeutic potential, as targeting mRNAs linked to AD risk or protection could lead to the development of novel treatments, including gene therapies aimed at modulating mRNA expression and potentially altering disease trajectories [54,55,56].

## 4. Materials and Methods

### 4.1. Participants

We recruited 30 participants (15 with a diagnosis of AD and 15 healthy controls) at the Instituto Colombiano de Neuropedagogía (ICN) in Barranquilla, Colombia. The ICN team determined the candidates’ eligibility based on the Montreal Cognitive Assessment (MoCA) results [58] and the inclusion criteria described elsewhere [13].

Patients were classified as affected by AD if they met the DSM-V criteria [59] and had a Mini-Mental State Examination (MMSE) [60] score between 0 and 18 points. Exclusion criteria included other neurological or major psychiatric disorders, psychoactive substance use, excessive alcohol consumption, and inability to complete the clinical studies as previously described [13]. Healthy controls were non-family volunteers over 65 years old, without suspected AD, and with an MMSE score between 19 and 29. Individuals with depression, mild cognitive impairment (MCI), dementia, other neurological disorders, major psychiatric illnesses, or those using psychoactive substances or consuming excessive alcohol were excluded.

### 4.2. Neuropsychological Assessment

After explaining to potential participants what the study consisted of and obtaining informed consent, an exhaustive neuropsychological evaluation was performed, which included the following tests: Boston Denomination Test [61,62], Rey–Osterrieth Complex Figure [63], Rey Auditory Verbal Learning Test (RAVLT) [64], Trail Making Test (TMT) [65,66], Symbol Digit Modality Test (SDMT) [67], Stroop Color and Word Test [68], Token Test [69], Benton’s Visual Retention Test (BVRT) [70], Clock Drawing Test [71], Memory Scale subtest of the Wisconsin Card Testing Test [72], Geriatric Depression Screening Test [73], Global Deterioration Scale (GDS) [74], Barthel Functional Index [75], and Neuropsychiatric Inventory [76]. Additional data for each participant, such as age at the beginning of the study, sex, educational level, marital status, weight, and height, were also recorded through the clinical history. In participants diagnosed with AD, the AD age of onset (ADAOO) of the disease was defined as the age at onset of symptoms according to previous research [77,78].

### 4.3. RNA Isolation and Extraction

Blood samples were collected to isolate circulating exosomes as described elsewhere [13]. Exosomes were isolated using the Total Exosome Isolation Reagent commercial kit (catalogue #4478360, Thermo Fisher Scientific, Inc., Waltham, MA, USA) following the manufacturer’s instructions with minor modifications standardized at Universidad del Norte, Barranquilla laboratories. The resulting exosomes were characterized by scanning electron microscopy (SEM). For this purpose, exosomes were encapsulated with nanodiamond particles, and their sizes were confirmed.

For the extraction of RNA contained in exosomes, a technique based on the acid phenol–chloroform method was standardized in the laboratory of the Universidad del Norte [13]. Extracted RNA was resuspended with 50 µL of RNAse-free water and then subjected to DNase I (catalogue #EN0521, Thermo Fisher Scientific, Inc., USA) following the manufacturer’s instructions. Finally, the concentration and indexes of the readings obtained with the optical densities (ODs) 260/230 and 260/280 were measured in a NanoDrop 2000 (Thermo Fisher Scientific, Inc., USA) and corresponded to the RNA quality indexes.

### 4.4. mRNA Microarray Study

For mRNA identification and differential expression analysis, the 30 RNA samples (15 cases with AD and 15 healthy controls) were sent to Arraystar, Inc. (Rockville, MD, USA), where RNA quality control, labelling, and hybridization were performed according to Agilent’s single-color microarray-based gene expression analysis protocol (Agilent Technologies, Santa Clara, CA, USA) with minor modifications.

#### 4.4.1. Quality Control

Each sample was subjected to retrotranscription to obtain complementary DNA (cDNA), amplified, and transcribed back to its complementary RNA (cRNA). In this step, amplification and incorporation of the cyanine 3 (Cy3) fluorescent dye labelling was achieved simultaneously along the entire length of the 3′ unbiased transcript using a random priming method (Arraystar Flash RNA Labelling Kit, Arraystar, Inc., Rockville, MD, USA). The labelled cRNAs were purified with the RNeasy mini kit (Qiagen, Hilden, Germany). In this step, reagent residues and the excess of cyanine not incorporated were eliminated. As a control of the amplification and labelling process of the samples, the concentration of the cRNA was obtained, and the rate of cyanine incorporation or specific activity (pmol of Cy3 per μg cRNA). Hybridization was allowed to continue if the cRNA concentration was >1.65 μg and the specific activity was >9 pmol of Cy3 per μg of cRNA. Otherwise, cRNA preparation was repeated.

#### 4.4.2. Hybridization and Microarray Scanning

A total of 1 μg of each labelled cRNA was fragmented by adding five μL of blocking agent 10x and 1 μL of fragmentation buffer 25x. The mixture was heated to 60 °C for 30 min, and then 25 μL of hybridization buffer 2x GE was used to dilute the labelled cRNA; 50 μL of hybridization solution was dispensed onto a hybridization plate, which was then assembled with an lncRNA expression microarray plate. The plates were incubated for 17 h at 65 °C in an Agilent hybridization oven. The hybridized arrays were washed and scanned using an Agilent scanner (equipment #G2505C, Agilent Technologies, Santa Clara, CA, USA).

#### 4.4.3. mRNA Microarray and Data Normalization

The Arraystar Human LncRNA Arrays V5 is designed to systematically profile long non-coding RNAs (lncRNAs) and the entire set of protein-coding mRNAs: about 39,317 lncRNAs (8393 Gold Standard LncRNAs and 30,924 Reliable LncRNAs) and 21,174 mRNA coding transcripts. Arraystar, Inc. maintains high-quality proprietary lncRNA transcriptome databases that extensively collect lncRNAs through all major public databases and repositories, knowledge-based mining of scientific publications, and our lncRNA discovery pipelines, which include FANTOM5 CAT (version 1), GENECODE (version 29), RefSeq (updated to November 2018), BIGTranscriptome (version 1), knownGene (updated to November 2018), lncRNAdb, LncRNAWiki, RNAdb, NRED, CLS FL, NONCODE (version 5), MiTranscriptome (version 2), and an lncRNA/mRNA discovery pipeline from more than 47 Tb RNA-seq data. Each transcript is represented by a specific exon or splice junction probe, which can identify individual transcripts accurately. Positive and negative probes for housekeeping genes were printed onto the array for hybridization quality control.

Quantile normalization and subsequent data processing were performed using the GeneSpring GX v12.1 software package (Agilent Technologies, Santa Clara, CA, USA). After normalization, mRNAs were flagged as present or marginal (“all-target value”) in at least 15 of 30 samples chosen for further analysis.

### 4.5. Identification of mRNAs Conferring Susceptibility to AD

mRNAs conferring susceptibility to AD were identified using Generalized Linear Models [79]. For the *j*th mRNA, a Logistic regression model of the form AD ~ mRNA*_j_* + Age + Sex + Schooling was fitted using the glm() function in R version 4.4.1 [80], where Age is the age of the individual at the beginning of the study and Schooling corresponds to years of education. Subsequently, we extracted the estimated regression coefficient associated with mRNA*_j_*, denoted as ${\hat{\beta }}_{j}$, the corresponding standard error ${\hat{SE}}_{{\hat{\beta }}_{j}}$ and the test statistic computed as $t_{j}=\frac{{\hat{\beta }}_{j}}{{\hat{SE}}_{{\hat{\beta }}_{j}}}$. For interpretation purposes, ${\hat{\beta }}_{j}>0$ implies that the *j*th mRNA confers susceptibility to AD; ${\hat{\beta }}_{j}<0$ implies that the *j*th mRNA has a protective effect; and ${\hat{\beta }}_{j}=0$ implies that the *j*th mRNA does not affect AD susceptibility (*j* = 1, 2, …, *m*). Under the null hypothesis, $t_{j}\sim t_{n-p}$. In our context, *n =* 30 and *p =* 5. The *p*-value for the *j*th mRNA is $P_{j}=2Pr(t_{25}>|t_{j}|)$. Thus, *p*-values $P_{1},\;P_{2},\;P_{3},\ldots ,P_{m}$ are collected. As *m* is usually large, *p*-values were corrected for multiple testing using the false discovery rate (FDR) [81,82]. mRNAs with FDR-corrected *p*-values below 5% (*p*_FDR_ < 0.05) were statistically significantly associated with AD susceptibility.

### 4.6. mRNA Differentially Expressed Between AD Groups

mRNAs differentially expressed between individuals with AD and healthy controls were identified using a Gamma regression model with an identity link of the form mRNA*_j_* ~ AD + Age + Sex + Schooling was fitted to the data as implemented in the glm() function of R. For the *j*th mRNA, the estimated regression coefficient associated with AD, denoted as ${\hat{\beta }}_{j}$, the standard error ${\hat{SE}}_{{\hat{\beta }}_{j}}$, and the test statistic $t_{j}$ were extracted (for more details, see Section 4.5). For interpretation purposes, ${\hat{\beta }}_{j}>0$ implies that the *j*th mRNA is upregulated in individuals with AD; ${\hat{\beta }}_{j}<0$ implies that the *j*th mRNA is downregulated; and ${\hat{\beta }}_{j}=0$ implies that there is no difference in the average expression levels of the *j*th mRNA between the comparison groups. The *p*-value of AD for the *j*th mRNA is calculated as $P_{j}=2Pr(t_{25}>|t_{j}|)$. Further, the collection of *p*-values $P_{1},\;P_{2},\;P_{3},\ldots ,{\;P}_{m}$ was corrected for multiple testing using FDR, with only those with *p*_FDR_ < 0.05 considered differentially expressed between individuals with AD and healthy controls.

### 4.7. mRNA Associated with ADAOO

mRNAs potentially associated with ADAOO were identified using a Gamma regression model of the form ADAOO ~ mRNA*_j_* + Age + Sex + Schooling with an identity link. Next, the regression coefficient associated with mRNA*_j_*, denoted as ${\hat{\beta }}_{j}$, as well as the standard error ${\hat{SE}}_{{\hat{\beta }}_{j}}$ and the test statistic $t_{j}$, were extracted. In this case, only individuals with AD were considered for analysis. For interpretation purposes, ${\hat{\beta }}_{j}>0$ implies that the *j*th mRNA delays ADAOO; ${\hat{\beta }}_{j}<0$ implies that the *j*th mRNA accelerates ADAOO; and ${\hat{\beta }}_{j}=0$ implies that the *j*th mRNA has no effect on ADA. The *p*-value for the *j*th mRNA is calculated as $P_{j}=2Pr(t_{10}>|t_{j}|)$. Therefore, mRNAs with *p*_FDR_ < 0.05 were associated with ADAOO.

### 4.8. Identification of mRNA Signatures Relevant to AD and ADAOO Using ML

We utilized the OneR package [83,84] in R to construct a simple and interpretable rule-based predictive model for AD and ADAOO. The OneR ML algorithm generates one-rule models for each predictor in the data and selects the single most predictive attribute for predicting an outcome variable of interest [83,84]. In this case, the mRNA expression levels were included as predictor variables, and the outcome variables were AD diagnosis (0: control; 1: case) and ADAOO. For each mRNA, OneR counts how often each class (AD diagnosis or a categorized version of ADAOO) appears, finds the most frequent class, makes a rule that assigns that class to the mRNA expression level, and calculates the error of that rule.

### 4.9. ML-Based Predictive Framework with mRNA Signatures

We used the caret package [85,86] in R to construct predictive models of AD status (0: control; 1: case) using the expression levels of mRNAs and demographic variables (i.e., age at the beginning of the study, sex, and years of education) as predictors. This package implements a series of ML algorithms and a comprehensive framework for building, testing, and validating ML models for classification and regression [85,86].

To develop ML models for AD, we employed several algorithms: Classification and Regression Tree (CART), Bagged CART, Random Forest (RF), XGBoost (xgbTree and xgbLinear), Support Vector Machines (SVMs), Linear Discriminant Analysis (lda), *K*-nearest Neighbors (knn), and Model Averaged Neural Network (avNNet). These algorithms were selected for their capacity to manage complex relationships in the data and deliver robust predictions. For details on these algorithms and their parameters, see Table S1 of the Supplementary Material. The dataset (*n* = 30) was partitioned into training (70%, *n* = 21) and testing (30%, *n* = 9) datasets. The performance of each algorithm was evaluated using accuracy metrics derived from the cross-validation process, which emphasizes showing the models’ predicted outcomes compared to actual results [87,88]. Each ML-based model was evaluated using the accuracy, the Receiver Operating Characteristic (ROC) curve, the area under the ROC curve (AUC), sensitivity, specificity and precision. These metrics assess how well the model predictions align with actual outcomes, with higher values indicating better performance [87,89].

We constructed an ML-based predictive framework for ADAOO. In addition to the ML algorithms previously mentioned, the performance of Ridge Regression (ridge), Generalized Linear Models (GLM), Generalized Additive Models (gam) using Locally Estimated Scatterplot Smoothing (LOESS; gamLoess), Multi-Layer Perceptron (mlp), and Partial Least Squares (pls) was also assessed (Supplementary Table S1). The original dataset (*n* = 15) was partitioned into training (*n* = 11) and testing (*n* = 4) datasets using the same proportions. As ADOO is a numerical variable, the performance of the ML-based models was assessed using the Mean Absolute Error (MAE), Mean Squared Error (MSE), and the coefficient of determination (*R*^2^). All models for AD diagnosis or ADAOO were trained using mRNA expression levels as predictors and utilized a 10-fold cross-validation procedure. This approach was specifically designed to ensure unbiased evaluations and enhance our understanding of how the models will likely perform on future unseen data.

## 5. Conclusions

Alzheimer’s disease (AD) is a progressive neurodegenerative disorder characterized by cognitive decline, primarily due to the accumulation of amyloid beta (Aβ) plaques and tau tangles in the brain [90]. Current diagnostic methods often rely on clinical assessments and imaging techniques. However, a growing interest in molecular biomarkers, particularly messenger RNA (mRNA) expression profiles, may enhance diagnostic accuracy and provide insights into the disease mechanisms in AD [15,17,91].

This study provides a framework for integrating ML and mRNA expression analysis, paving the way for personalized medicine approaches in AD. By identifying specific mRNAs associated with AD diagnosis and age of onset (ADAOO), we contribute to a deeper understanding of the biological underpinnings of AD and its progression.

Our findings reveal that after false discovery rate (FDR) correction, only ENST00000331581 (*CADM1*) and ENST00000382258 (*TNFRSF19*) were statistically significantly differentially expressed between the comparison groups (Table 3). In addition, ENST00000340552 (*LIMK2*) was strongly associated with Alzheimer’s disease age of onset (ADAOO), accelerating AD onset by approximately 12.6 years (Table 4). Based on machine learning (ML) algorithms, the researchers identified that the expression levels of ENST00000331581 (*CADM1*), ENST00000372572 (*FOXJ3*), and ENST00000311550 (*GABRB3*) achieved an accuracy of 95.4% for predicting AD diagnosis (Table 5). Similarly, the expression levels of ENST00000640218 (*HNRNPU*), ENST00000261245 (*MNAT1*), and ENST00000339562 (*NR4A2*) showed remarkable performance in accurately predicting ADAOO (Table 5).

The use of ML algorithms combined with mRNA expression data offers a promising avenue for early diagnosis and personalized treatment strategies. Here, we further investigated the predictive power of various ML algorithms for predicting AD diagnosis and ADAOO based on mRNA expression. ENST00000311550 (*GABRB3*) emerged as the most significant predictor for AD diagnosis, and additional mRNAs—ENST00000278765 (*GGTLC1*), ENST00000331581 (*CADM1*), ENST00000372572 (*FOXJ3*), and ENST00000636358 (*ACY1*)—were critical predictors of AD diagnosis (Supplementary Figure S1). Notably, these mRNAs demonstrated exceptional performance, distinguishing individuals with AD from healthy controls (Supplementary Figure S1). For predicting ADAOO, ENST00000304677 (*RNASE6*) and ENST00000602017 (*PPP5D1*) have achieved the best performance metrics in the testing data, suggesting that the prediction error for ADAOO is limited to a few months (Table 9).

While our findings are promising, this study has several limitations regarding population characteristics and sample diversity. First, the relatively small sample size may restrict the generalizability of the results, requiring further validation in larger, more diverse cohorts to confirm the reliability of these biomarkers and their roles in AD. Second, the stringent inclusion and exclusion criteria may create a homogeneous sample that does not adequately represent the variability of AD in the broader population, including different clinical subtypes. Lastly, individual variability in disease progression and comorbid conditions can mask treatment effects and challenge data interpretation.

Future research should focus on several key areas to advance our understanding of AD. First, conducting larger-scale studies is essential to validate the identified mRNA biomarkers across diverse populations, ensuring their robustness and applicability in clinical settings. Second, exploring the biological roles of *CADM1*, *TNFRSF19*, and *LIMK2* through functional studies will help elucidate their contributions to neurodegenerative processes and AD pathogenesis. Finally, investigating potential therapeutic strategies aimed at modulating the expression of these mRNAs or targeting their associated pathways could provide innovative approaches to delay or prevent the onset of AD. By focusing on these areas, future studies can significantly enhance diagnostic accuracy and therapeutic interventions for AD.

In summary, this study highlights the potential of ML combined with exosomal mRNA expression analysis in advancing the understanding of AD. The identified mRNA transcripts and the robust predictive models developed offer a promising avenue for more accurate and early diagnosis, ultimately leading to improved patient outcomes. Continued refinement of these models and further investigation into the underlying biological mechanisms will be crucial in translating these findings into clinical practice and driving therapeutic innovations.

## Figures and Tables

**Table 1:** Clinical and sociodemographic characterization of the study population.

Variable	All (*n* = 30)	Cases (*n* = 15)	Controls (*n* = 15)	*p*
	Mean (SD)			
Age (years)	79.8 (8.7)	77.5 (8.5)	82.1 (8.6)	0.261
Age of onset (years)	72.1 (7.2)	72.1 (7.2)	-	*-*
MMSE	19.6 (9.6)	13.9 (9.5)	25.2 (5.6)	0.001
MoCA	15.3 (11.2)	5.5 (5.3)	25.9 (3)	<0.001
	Frequency (%)			
Sex				1
Female	22 (73.3%)	11 (73.3%)	11 (73.3%)	
Male	8 (26.7%)	4 (26.7%)	4 (26.7%)	

MMSE: Mini-Mental State Examination; MoCA: Montreal Cognitive Assessment; SD: standard deviation; *p*: *p*-value.

**Table 2:** Top 10 mRNAs conferring susceptibility to AD.

Chr	Transcript ID	Position *^a^*	Gene	$\hat{\beta }\;({\hat{SE}}_{\hat{\beta }})$	*p*	*p*_FDR_
11	ENST00000382160	1,718,425	*KRTAP5-6*	5.27 (1.97)	0.007	0.999
11	MICT00000062561	68,830,976	*TPCN2*	2.74 (1.03)	0.007	0.999
2	ENST00000272252	38,893,052	*GALM*	3.18 (1.20)	0.008	0.999
19	ENST00000263372	38,810,484	*KCNK6*	4.52 (1.71)	0.008	0.999
11	ENST00000292174	118,754,475	*CXCR5*	3.76 (1.45)	0.009	0.999
19	ENST00000601440	20,802,867	*ZNF626*	2.38 (0.92)	0.009	0.999
2	ENST00000406226	48,757,325	*STON1*	3.74 (1.46)	0.010	0.999
3	ENST00000318225	126,268,516	*C3orf22*	7.84 (3.07)	0.010	0.999
9	ENST00000307564	117,096,436	*AKNA*	2.34 (0.92)	0.010	0.999
17	ENST00000537494	73,632,675	*SMIM5*	2.16 (0.85)	0.010	0.999

*^a^* UCSC GRCh37/hg19 coordinates. $\hat{\beta }$: logistic regression coefficient; Chr: chromosome; FDR: false discovery rate; *p*: *p*-value; *p*_FDR_: FDR-corrected *p*-value; ${\hat{SE}}_{\hat{\beta }}$: estimated standard error of $\hat{\beta }$.

**Table 3:** Top 10 mRNAs differentially expressed between cases and healthy controls.

Chr	Transcript	Position	Gene	$\hat{\beta }\;({\hat{SE}}_{\hat{\beta }})$	*p*	*p*_FDR_
11	ENST00000331581	115,047,015	*CADM1*	0.97 (0.16)	3.34 × 10^−6^	0.027
13	ENST00000382258	24,153,499	*TNFRSF19*	0.40 (0.06)	2.24 × 10^−6^	0.027
3	ENST00000318225	126,268,516	*C3orf22*	0.71 (0.14)	2.32 × 10^−5^	0.128
17	ENCT00000175321	42,030,339	*PYY*	0.82 (0.18)	1.74 × 10^−4^	0.692
19	ENST00000358491	21,688,366	*ZNF429*	0.83 (0.19)	2.16 × 10^−4^	0.692
2	ENST00000406226	48,757,325	*STON1*	0.72 (0.17)	2.50 × 10^−4^	0.692
19	ENST00000263372	38,810,484	*KCNK6*	0.82 (0.20)	3.52 × 10^−4^	0.833
7	ENCT00000407904	1,214,597	*ZFAND2A*	0.60 (0.15)	6.07 × 10^−4^	0.985
1	ENST00000427500	155,204,350	*GBA*	0.83 (0.22)	7.12 × 10^−4^	0.985
5	ENST00000509437	132,333,792	*ZCCHC10*	0.72 (0.18)	6.33 × 10^−4^	0.985

$\hat{\beta }$: Gamma regression coefficient based on the identity link. Other conventions as in Table 2.

**Table 4:** mRNAs modifying ADAOO. Conventions as in Table 3.

Chr	Transcript	Position	Gene	$\hat{\beta }\;({\hat{SE}}_{\hat{\beta }})$	*p*	*p*_FDR_
22	ENST00000340552	31,644,473	*LIMK2*	−12.6 (1.06)	3.04 × 10^−7^	0.005
22	ENST00000215730	21,213,271	*SNAP29*	−5.59 (0.76)	2.50 × 10^−5^	0.096
22	ENST00000216139	51,176,624	*ACR*	−7.21 (1.27)	2.14 × 10^−4^	0.096
5	ENST00000230658	50,679,225	*ISL1*	−11.05 (1.49)	2.29 × 10^−5^	0.096
4	ENST00000248706	53,728,457	*RASL11B*	−6.18 (1.09)	2.15 × 10^−4^	0.096
7	ENST00000257696	128,095,945	*HILPDA*	4.34 (0.76)	2.00 × 10^−4^	0.096
8	ENST00000263851	79,645,007	*IL7*	−17.31 (2.96)	1.61 × 10^−4^	0.096
13	ENST00000282397	28,874,481	*FLT1*	−6.21 (1.01)	1.07 × 10^−4^	0.096
19	ENST00000304060	11,925,099	*ZNF440*	4.79 (0.79)	1.15 × 10^−4^	0.096
3	ENST00000320211	48,488,137	*ATRIP*	−6.93 (1.08)	7.47 × 10^−4^	0.096

**Table 5:** Top mRNAs for AD diagnosis and ADAOO via ML in the training dataset.

Target Variable	Chr	Transcript	Position	Gene	Accuracy
AD	11	ENST00000331581	115,047,015	*CADM1*	0.954
	1	ENST00000372572	42,642,210	*FOXJ3*	0.954
	15	ENST00000311550	26,788,693	*GABRB3*	0.954
	17	ENST00000293190	72,838,162	*GRIN2C*	0.904
	21	ENST00000311124	46,933,690	*SLC19A1*	0.904
	2	MICT00000202802	171,678,607	*GAD1*	0.904
	3	ENCT00000296543	161,062,306	*SPTSSB*	0.904
	1	ENST00000427500	155,204,350	*GBA*	0.904
	16	ENST00000571688	11,641,578	*LITAF*	0.904
	3	ENST00000636358	52,017,294	*ACY1*	0.904
ADAOO	1	ENST00000640218	245,013,602	*HNRNPU*	1.000
	14	ENST00000261245	61,201,480	*MNAT1*	1.000
	2	ENST00000339562	157,180,944	*NR4A2*	1.000
	14	ENST00000304677	21,249,210	*RNASE6*	1.000
	2	ENST00000263736	45,615,819	*SRBD1*	1.000
	17	ENST00000394001	39,533,902	*KRT34*	0.900
	3	ENST00000264735	192,958,914	*HRASLS*	0.900
	20	ENCT00000265279	20,349,595	*INSM1*	0.900
	8	ENST00000313269	145,064,226	*GRINA*	0.900
	5	ENST00000257430	112,073,585	*APC*	0.900

**Table 6:** Performance of ML-based models for AD diagnosis in the training dataset. Best results are shown in **bold**.

Algorithm	Accuracy		
	Mean	Standard Deviation	Coefficient of Variation
avNNet	0.780	0.237	30.354
hdda	0.783	0.243	31.072
knn	0.783	0.234	29.858
LDA	0.857	0.238	27.796
lda2	0.857	0.238	27.796
rf	**0.947**	**0.148**	**15.683**
rpart	0.847	0.295	34.862
rpart1SE	0.847	0.295	34.862
rpart2	0.847	0.295	34.862
svmLinear	0.787	0.238	30.278
svmLinear2	0.787	0.238	30.278
svmPoly	0.820	0.228	27.802
svmRadial	0.807	0.227	28.113
treebag	0.927	0.224	24.147
xgbLinear	**0.980**	**0.141**	**14.431**
xgbTree	**0.990**	**0.071**	**7.142**

**Table 7:** Performance metrics for predicting AD diagnosis based on the xgbTree algorithm.

Performance Metric	Dataset	
	Training (*n* = 21)	Testing (*n* = 9)
AUC	1	0.875
Accuracy	1	0.875
Sensitivity	1	1
Specificity	1	0.75
Precision	1	1

**Table 8:** Performance of ML algorithms for predicting ADAOO based on the top 30 mRNAs.

Algorithm	Performance Measure		
	RMSE	*R* ^2^	MAE
avNNet	71.518	-	71.510
gamLoess	29.955	1	28.094
glm	29.955	1	28.094
**knn**	**6.817**	1	**6.761**
mlp	7.606	-	7.530
**pls**	**6.519**	1	**6.459**
rf	7.227	1	7.190
ridge	7.834	1	7.802
rpart	7.576	-	7.497
rpart1SE	7.576	-	7.497
svmLinear	10.067	1	10.060
svmPoly	6.969	1	6.887
svmRadial	7.234	1	7.168
treebag	7.587	-	7.506
xgbLinear	10.306	1	10.235
xgbTree	8.250	1	8.155

RMSE: Root Mean Squared Error, lower is better; MAE: Mean Absolute Error, lower is better; *R*^2^: coefficient of determination, higher is better. “-” indicates that *R*^2^ values could not be estimated. Best results are shown in **bold**.

**Table 9:** Performance of refined rf, xgbLinear, and xgbTree ML models for predicting ADAOO in the testing data. Best results are shown in **bold**.

Algorithm	Model	mRNAs Combination	RMSE	*R* ^2^	MAE
rf	1	HBMT00001385713, ENCT00000265279	2.701	0.743	2.156
	2	HBMT00001385713, ENST00000370332	1.698	0.894	1.569
	3	HBMT00001385713, ENST00000257430	**0.974**	**0.975**	**0.840**
xgbLinear	1	HBMT00001385713, ENST00000263736	3.484	0.656	1.747
	2	HBMT00001385713, ENST00000304677	5.500	0.303	2.750
	3	HBMT00001385713, ENST00000640218	**2.554**	**0.815**	**1.278**
xgbTree	1	ENST00000304677, ENST00000640218	1.564	0.979	1.218
	2	ENST00000304677, ENST00000602017	**0.462**	**0.993**	**0.392**
	3	ENST00000304677, ENST00000224950	0.740	0.999	0.613
	4	ENST00000304677, ENST00000322088	2.054	0.983	1.719

## Data Availability

The data presented in this study are available upon reasonable request from the corresponding authors. They are not publicly available due to the ongoing nature of the study and our commitment to protecting the privacy and confidentiality of our patients.

## Supplementary Materials

The supporting information can be downloaded at https://www.mdpi.com/article/10.3390/ijms252212293/s1.
