# Supplementary material for: Exosomal mRNA Signatures as Predictive Biomarkers for Risk and Age of Onset in Alzheimer’s Disease
Source: Int J Mol Sci. 2024 Nov 15;25(22):12293. doi: 10.3390/ijms252212293 (PMC11594294; doi:10.3390/ijms252212293)
Supplement: Supplementary file 1 [file ijms-25-12293-s001.zip › ijms-3228078-supplementary.pdf]

International Journal of
Molecular Sciences

MDPI

Supplementary Material

Exosomal mRNA signatures as Predictive Biomarkers for Risk and Age of Onset in Alzheimer's disease

Daniel A. Bolívar [1,*], María I. Mosquera-Heredia [2,*], Oscar M. Vidal [2,*], Ernesto Barceló [3,4,5], Ricardo Allegri [6], Luis C. Morales [2], Carlos Silvera-Redondo [2], Mauricio Arcos-Burgos [7], Pilar Garavito-Galofre [2,†,#], Jorge I. Vélez [1,†,#]

[1] Department of Industrial Engineering, Universidad del Norte, Barranquilla 081007, Colombia.
[2] Department of Medicine, Universidad del Norte, Barranquilla 081007, Colombia.
[3] Instituto Colombiano de Neuropedagogía, Barranquilla 080020, Colombia.
[4] Department of Health Sciences, Universidad de La Costa, Barranquilla 080002, Colombia.
[5] Grupo Internacional de Investigación Neuro-Conductual (GIINCO), Universidad de La Costa, Barranquilla 080002, Colombia.
[6] Institute for Neurological Research FLENI, Montañeses 2325, Buenos Aires C1428AQK, Argentina.
[7] Grupo de Investigación en Psiquiatría (GIPSI), Departamento de Psiquiatría, Instituto de Investigaciones Médicas, Facultad de Medicina, Universidad de Antioquia, Medellín 050010, Colombia.
* Correspondence: mpgaravi@uninorte.edu.co (P.G.-G.); jvelezv@uninorte.edu.co (J.I.V.)
† These authors contributed equally to this work.

Citation: Bolívar, D.A., Mosquera-Heredia, M.I.; Vidal, O.M.; Barceló, E.; Allegri, R.; Morales, L.C.; Silvera-Redondo, C.; Arcos-Burgos, M.; Garavito-Galofre, P; Vélez, J.I. mRNA signatures predict risk and age of onset in Alzheimer's disease. *Int. J. Mol. Sci.* **2024**, *25*, x. https://doi.org/10.3390/xxxxx

Academic Editor: Firstname Lastname

Received: date
Revised: date
Accepted: date
Published: date

Copyright: © 2023 by the authors. Submitted for possible open access publication under the terms and conditions of the Creative Commons Attribution (CC BY) license (https://creativecommons.org/licenses/by/4.0/).

Table S1. ML algorithms used to predict Alzheimer's disease (AD) diagnosis and AD age of onset (ADAOO).

Algorithm	Method[a]	Type	Description
Classification and Regression Tree (CART)	rpart, rpart2, rpart1SE	Classification and Regression	Non-parametric method that recursively splits data into homogeneous subgroups based on predictive variables.
Bagged CART	treebag	Classification and Regression	Combines multiple CART models to improve prediction accuracy and reduce overfitting.
Random Forest (RF)	rf	Classification and Regression	Combines multiple decision trees, using random feature subsets, to improve prediction accuracy and reduce overfitting.
Extreme Gradient Boosting (XGBoost)	xgbTree , xgbLinear	Classification and Regression	Uses gradient boosting to combine multiple decision trees, improving prediction accuracy and reducing overfitting.
Support Vector Machines (SVM)	svmLinear, svmLinear2, svmPoly, svmRadial	Classification and Regression	Supervised learning algorithm that classifies data by transforming it into a higher-dimensional space using kernel functions.
Linear Discriminant Analysis (L	lda, lda2	Classification and Regression	Linear method that projects high-dimensional data onto a lower-dimensional space to enhance classification accuracy.
K-nearest neighbors (KNN)	knn	Classification and Regression	Non-parametric method that classifies data based on the majority vote of its nearest neighbors in feature space.
Ridge Regression (ridge)	ridge	Regression	The linear regression method mitigates multicollinearity by adding a penalty term to the loss function.
Generalized Linear Model (GLM)	glm	Regression	Linear model that accommodates non-normal data by employing a link function to map data to a linear space.
Generalized Additive Model using LOESS	gamLoess	Regression	A non-parametric method that models relationships between variables using a combination of linear and non-linear components.
Multi-Layer Perceptron	mlp	Classification and Regression	Neural network model with multiple layers of interconnected nodes that learns intricate input-output relationships.
Partial Least Squares	pls	Regression	Method that blends regression and dimensionality reduction to model relationships between variables.
Model Averaged Neural Network	avNNet	Classification and Regression	Technique that combines multiple neural network models to enhance prediction accuracy and reduce overfitting.

[a] Refers to the string used to for estimating a particular ML algorithm via the caret package in R. The complete list of ML algorithms implemented in caret is available at https://topepo.github.io/caret/available-models.html.

Table S2. Top 30 mRNAs for predicting AD diagnosis based on the OneR ML algorithm.

Transcript	Chr	Position	Gene Symbol	Accuracy
ENST00000331581	11	115047015	*CADM1*	0.954
ENST00000372572	1	42642210	*FOXJ3*	0.954
ENST00000311550	15	26788693	*GABRB3*	0.954
ENST00000293190	17	72838162	*GRIN2C*	0.904
ENST00000311124	21	46933690	*SLC19A1*	0.904
MICT00000202802	2	171678607	*CATG00000050657.1*	0.904
ENCT00000296543	3	161062306	*CATG00000062558.1*	0.904
ENST00000427500	1	155204350	*GBA*	0.904
ENST00000571688	16	11641578	*LITAF*	0.904
ENST00000636358	3	52017294	*ACY1*	0.904
ENST00000278765	20	23965690	*GGTLC1*	0.904
HBMT00001159081	5	13967518	*CATG00000079193.1*	0.904
ENST00000296721	5	148651434	*AFAP1L1*	0.904
ENST00000640345	16	81055921	*AC092718.8*	0.904
ENST00000358491	19	21688366	*ZNF429*	0.904
ENST00000391945	19	45853095	*ERCC2*	0.904
ENST00000361115	5	43379295	*CCL28*	0.904
ENCT00000466207	X	40014392	*CATG00000110960.1*	0.904
ENST00000272647	2	128619204	*AMMECR1L*	0.857
ENST00000372441	6	43474707	*LRRC73*	0.857
ENCT00000176029	17	46100960	*CATG00000031631.1*	0.857
ENST00000409115	2	232573219	*PTMA*	0.857
ENST00000403665	4	187187099	*F11*	0.857
ENST00000215659	22	50691332	*MAPK12*	0.857
ENST00000403906	19	52839561	*ZNF610*	0.857
ENST00000223210	7	149535456	*ZNF862*	0.857
NM_001300856	11	65659607	*FOSL1*	0.857
ENST00000337979	11	59522532	*STX3*	0.857
HBMT00000553098	16	89772558	*CATG00000028352.1*	0.857
ENST00000210633	10	102732592	*SEMA4G*	0.857

Chr: Chromosome.

Table S3. Top 30 mRNAs for predicting ADAOO based on the OneR ML algorithm.

Transcript	Chr	Position	Gene Symbol	Accuracy
ENST00000640218	1	245013602	*HNRNPU*	1.000
ENST00000261245	14	61201480	*MNAT1*	1.000
ENST00000339562	2	157180944	*NR4A2*	1.000
ENST00000304677	14	21249210	*RNASE6*	1.000
ENST00000263736	2	45615819	*SRBD1*	1.000
ENST00000394001	17	39533902	*KRT34*	0.900
ENST00000264735	3	192958914	*HRASLS*	0.900
ENCT00000265279	20	20349595	*INSM1*	0.900
ENST00000313269	8	145064226	*GRINA*	0.900
ENST00000257430	5	112073585	*APC*	0.900
ENST00000373880	10	60936350	*PHYHIPL*	0.900
ENST00000310942	17	77751931	*CBX2*	0.900
ENST00000299320	16	4784273	*C16orf71*	0.900
ENST00000567659	16	29823512	*PRRT2*	0.900
ENST00000374531	9	112403068	*PALM2*	0.900
ENST00000539282	12	24970415	*BCAT1*	0.900
ENST00000602017	19	46984053	*PPP5D1*	0.900
ENST00000379663	4	122722475	*EXOSC9*	0.900
ENST00000359035	10	135122445	*ZNF511*	0.900
ENST00000262891	19	45754516	*MARK4*	0.900
ENST00000374198	9	116037922	*PRPF4*	0.900
ENST00000322088	19	52693292	*PPP2R1A*	0.900
ENST00000224950	10	105642300	*STN1*	0.900
ENST00000517427	8	80948820	*TPD52*	0.900
ENST00000220420	15	43524793	*TGM5*	0.900
ENST00000367474	6	147830063	*SAMD5*	0.900
ENST00000580887	18	3252273	*MYL12A*	0.900
ENST00000224337	10	97951458	*BLNK*	0.900
ENST00000370332	1	92940319	*GFI1*	0.900
HBMT00001385713	8	12612094	*LONRF1*	0.900

Chr: Chromosome.

Table S4. Assessment of ML models for AD diagnosis in the training data using the top 5 mRNAs as predictors.

**Algorithm**	**Accuracy**		
	**Mean**	**Standard Deviation**	**Coefficient of Variation**
avNNet	1.000	0.000	0.000
lda	1.000	0.000	0.000
lda2	1.000	0.000	0.000
svmLinear	1.000	0.000	0.000
svmLinear2	1.000	0.000	0.000
svmPoly	1.000	0.000	0.000
treebag	1.000	0.000	0.000
**xgbLinear**	**1.000**	**0.000**	**0.000**
**xgbTree**	**1.000**	**0.000**	**0.000**
svmRadial	0.963	0.127	13.213
knn	0.960	0.137	14.273
rf	0.950	0.152	15.949
hdda	0.947	0.148	15.682
rpart	0.867	0.269	31.081
rpart1SE	0.867	0.269	31.081
rpart2	0.867	0.269	31.081

a

Performance measure	Data set	
	Training (*n*=21)	Testing (*n*=9)
AUC	1	0.875
Accuracy	1	0.875
Sensitivity	1	1
Specificity	1	0.75
Precision	1	1

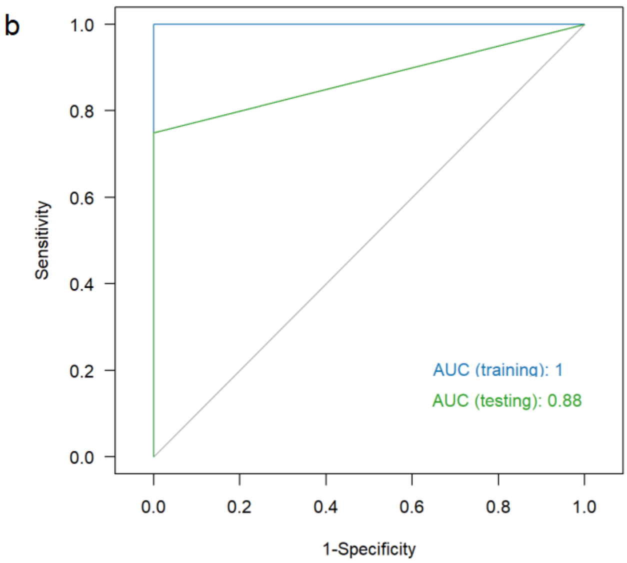

Figure S1. (**a**) Performance metrics and **(b)** ROC curves in the training (blue) and testing (green) datasets when predicting AD diagnosis based on the expression of ENST00000311550, ENST00000278765, ENST00000331581, ENST00000372572, and ENST00000636358 mRNAs using the xgbTree algorithm. Conventions as in Figure 3.

a

Combination	mRNAs	Accuracy (95% CI)
1	ENST00000311550-ENST00000372572	0.943 (0.884, 1)
2	ENST00000311550-ENST00000331581	0.958 (0.901, 1)
3	ENST00000311550-ENST00000278765	0.952 (0.887, 1)
4	ENST00000372572-ENST00000331581	0.845 (0.749, 0.941)
5	ENST00000372572-ENST00000278765	0.847 (0.769, 0.926)
6	ENST00000331581-ENST00000278765	0.878 (0.786, 0.969)
[illegible]	[illegible]	[illegible].95 (0.876, 1)
[illegible]	[illegible]	[illegible]17 (0.845, 0.988)

b

Performance measure	Data set	
	Training (*n*=21)	Testing (*n*=9)
AUC	1	1
Accuracy	1	1
Sensitivity	1	1
Specificity	1	1
Precision	1	1

Figure S2. (**a**) Accuracy and 95% confidence interval (CI) in the training data set, and (**b**) performance metrics for the best mRNA combination for predicting AD diagnosis using the xgbTree algorithm.

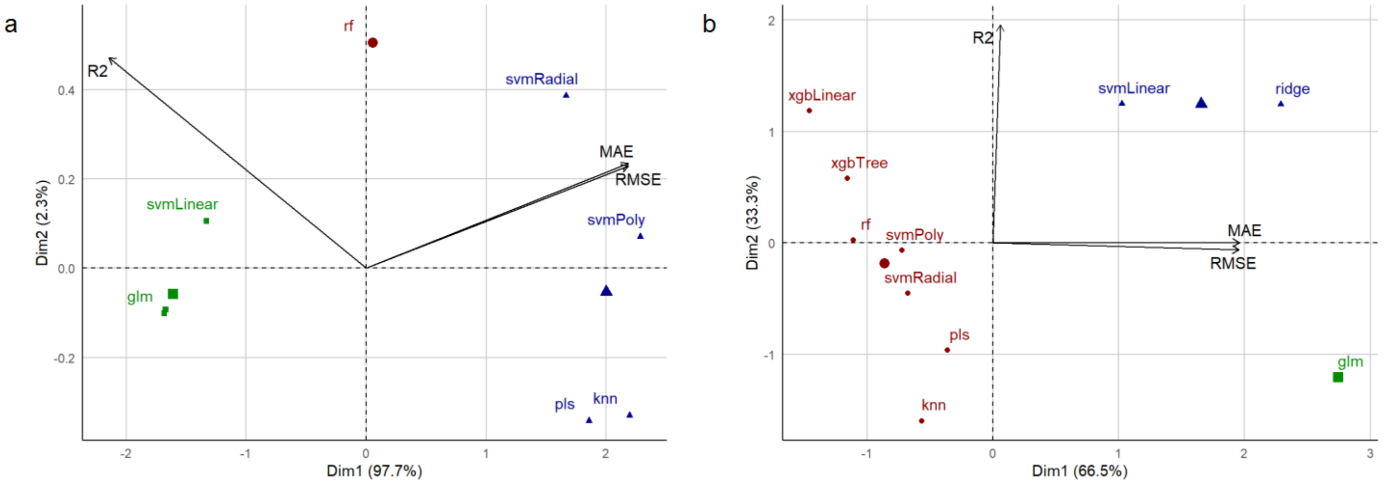

Figure S3. (**a**) PCA and *K*-means clustering representation of the performance measures for ML algorithms predicting ADAOO in the **(a)** training and **(b)** testing data sets. In the training dataset, Class 1 (green, Figure S3a) includes xgbLinear, xgbTree, and svmLinear, which consistently demonstrated superior performance; Class 2 (Figure S3a, Supplementat) includes only rf, which showed moderate performance; and Class 3 (blue, Figure S3a) includes pls, knn, svmPoly, and svmRadial, which exhibited varying degrees of accuracy and robustness, trailing classes 1 and 2. For the testing dataset, Class 1 (red, Figure S3b) includes glm, which performed poorly; Class 2 (blue, Figure S3b) includes ridge and svmLinear, showing modest predictive power; and Class 3 (green, Figure S3b) includes knn, pls, svmRadial, svmPoly, rf, xgbTree, and xgbLinear, which demonstrate competitive performance but varying outcomes compared to their training counterparts, indicating sensitivity to data distribution changes.
